# WOMen profEssioNal developmenT oUtcome Metrics in Academic Emergency Medicine: Results from the WOMENTUM Modified Delphi Study

**DOI:** 10.5811/westjem.2022.6.56608

**Published:** 2022-09-12

**Authors:** Jennifer S. Love, Amy J. Zeidan, Utsha G. Khatri, Margaret E. Samuels-Kalow, Angela M. Mills, Cindy H. Hsu

**Affiliations:** * Icahn School of Medicine at Mount Sinai, Department of Emergency Medicine, New York, New York; † Emory University School of Medicine, Department of Emergency Medicine, Atlanta, Georgia; ‡ Icahn School of Medicine at Mount Sinai, Department of Population Health Science and Policy, New York, New York; § Harvard Medical School, Massachusetts General Hospital, Department of Emergency Medicine, Boston, Massachusetts; ¶ Columbia University College of Physicians and Surgeons, Department of Emergency Medicine, New York, New York; || University of Michigan Medical School, Department of Emergency Medicine, Ann Arbor, Michigan

## Abstract

**Introduction:**

To address persistent gender inequities in academic medicine, women professional development groups (PDG) have been developed to support the advancement of women in medicine. While these programs have shown promising outcomes, long-term evaluative metrics do not currently exist. The objective of this study was to establish metrics to assess women’s PDGs.

**Methods:**

This was a modified Delphi study that included an expert panel of current and past emergency department (ED) chairs and Academy for Women in Academic Emergency Medicine (AWAEM) presidents. The panel completed three iterative surveys to develop and rank metrics to assess women PDGs. Metrics established by the expert panel were also distributed for member-checking to women EM faculty.

**Results:**

The expert panel ranked 11 metrics with high to moderate consensus ranking with three metrics receiving greater than 90% consensus: gender equity strategy and plan; recruitment; and compensation. Members ranked 12 metrics with high consensus with three metrics receiving greater than 90% consensus: gender equity strategy and plan; compensation; and gender equity in promotion rates among faculty. Participants emphasized that departments should be responsible for leading gender equity efforts with PDGs providing a supportive role.

**Conclusion:**

In this study, we identified metrics that can be used to assess academic EDs’ gender equity initiatives and the advisory efforts of a departmental women’s PDG. These metrics can be tailored to individual departmental/institutional needs, as well as to a PDG’s mission. Importantly, PDGs can use metrics to develop and assess programming, acknowledging that many metrics are the responsibility of the department rather than the PDG.

## INTRODUCTION

Gender disparities in academic medicine continue to exist in several areas including advancement, promotion, compensation, grant funding, and authorship.^1^–^6^ In response, dedicated programmatic interventions including mentorship programs, career development initiatives, and women’s professional development groups (PDG) have been created to target inequities and support the advancement of women in medicine. PDGs and similar gender equity programs have been associated with positive outcomes related to retention, advancement, and promotion of women in academic medicine.^7^–^10^

As institutional women’s PDGs grow in number, establishing a robust outcome assessment can help measure impact, support improvements, and ensure sustainability. While PDGs report positive outcomes and participant satisfaction, these studies have highlighted the need for long-term evaluative metrics.^7^,^11^ Various metrics have been used to describe PDG successes. For example, following PDG and workshop implementation, one institution reported an increased number of women faculty at all departmental rank levels.^12^ Other programs have described higher participant retention and career satisfaction, and development of gender-specific policies.^11^,^13^,^14^ Notably, participation in a national emergency medicine (EM) women’s PDG was associated with increased scholarly collaborations and mentorship/sponsorship that promoted participant visibility through speaking, leadership, and awards.^15^ While programs should be lauded for their various success, standard metrics for uniform PDG evaluation will allow cross-program comparison and strategic development of new programs.

In this study we developed measurable outcome metrics for departmental women’s PDGs using expert consensus from a panel of emergency department (ED) chairs and gender equity leaders in EM. Our goal in this study was to establish metrics to guide departmental PDG development and evaluation strategies.

## METHODS

### Study Design

We used modified Delphi methodology to establish metrics for women’s departmental PDG assessment. This methodology is widely accepted and commonly used to establish consensus from individuals with expertise specific to the desired topic.^16^–^19^ The Delphi technique uses sequential questionnaires to obtain opinions and agreement from participants on a topic where well-established consensus does not exist.^20^,^21^

### Study Participants

Expert panel participants met one of the following criteria: 1) current ED chair; 2) past ED chair; 3) current president of the Society for Academic Emergency Medicine’s (SAEM) Academy for Women in Academic Emergency Medicine (AWAEM); 4) past AWAEM president. We selected current/past ED chairs for their role in overseeing departmental activities including funding dissemination, diversity and equity initiatives, and career advancement. Current/past AWAEM presidents were selected for their expertise in recruitment, advancement, and leadership of women in EM. We recruited current/past ED chairs from the Association of Academic Chairs of Emergency Medicine (AACEM), while current/past AWAEM presidents were identified from the AWAEM website and contacted via email. We recruited current/past department chairs and current/past AWAEM presidents independently from their academic institutions. A single institution could have multiple participants.

**Population Health Research Capsule**

What do we already know about this issue?

*Women’s professional development groups (PDG) support the advancement of women in medicine, but no long-term evaluative metrics for PDGs exist*.

What was the research question?

*Based on consensus from emergency department (ED) chairs and gender equity leaders, what are the optimal evaluative metrics for women’s PDGs in emergency medicine?*

What was the major finding of the study?

*High-consensus women’s PDG metrics include workplace gender equity, compensation, recruitment, retention, and leadership*.

How does this improve population health?

*While many gender equity metrics are departmental responsibilities, women’s PDGs can use these metrics to guide programmatic development*.

Member-checking participants were recruited using the AWAEM and FemInEM (www.feminem.org) email listservs. Member-checking is a technique used to enhance the validity of metrics identified by experts.^22^ These listservs were selected because 1) their memberships include a large, diverse population of EM women faculty in the United States, and 2) their members include individuals who would likely participate in women’s PDGs.

### Delphi Procedure (Figure 1)

This study included four phases during which experts completed three questionnaires. In phase 1, participants completed an open-ended questionnaire to gather all relevant opinions. In phase 2, participants ranked summarized opinions from phase 1. In phase 3, participants ranked metrics with moderate and high consensus. In the final phase, we employed member-checking. Members reviewed and ranked phase 2 metrics. Member-checking supported the credibility of findings, acknowledging that members would most benefit from PDGs and would provide critical feedback on specific metrics. This study was reviewed and approved by the institutional review board at Oregon Health & Science University.

#### Phase 1: Qualitative Assessment

Expert participants completed an open-ended online questionnaire (“What metrics are important to assess for the effectiveness of a women’s PDG? Please name, describe, and give your reasoning for at least three metrics”), which solicited metrics to evaluate women PDGs. Participants were asked to list metrics to evaluate women’s PDGs, a metric description, and a rationale for why metric inclusion was important. Responses were manually reviewed and qualitatively analyzed by three authors (JL, AZ, UK) using an iterative approach until consensus on thematic categorizations was achieved.^23^ We then categorized common metrics thematically.

#### Phase 2: All Metrics Ranking Survey

We developed a ranking survey (survey 2) using phase 1 responses, which we sent to all initial expert participants. The survey included each metric and assessment methods for the individual metric. Sub-metrics were included for some metrics. Participants ranked metrics by level of importance using a five-point priority scale. They were provided the following prompt: “*Your department’s women’s professional development group (PDG) requests funding and support for the upcoming academic year. What metrics should the PDG measure to determine the success of the program? Please categorize the metrics listed below as lowest (1) to highest (5) priority in evaluating the PDG to decide whether or not you contribute funding support*.” The study question was framed around PDG funding support because departmental support for specialized interests (ie, research, operations, education) is frequently provided as financial or time support.

We analyzed responses using high, moderate, and low consensus. Consensus was defined as the degree to which participants agreed on metrics. Consensus was considered to be high if there was >80% agreement in two contiguous categories (priority score 4 or 5) and moderate consensus was considered 70–80% agreement in two contiguous categories.^24^ Low consensus was considered <70% agreement in two contiguous categories.

#### Phase 3: Ranking Survey

The phase 3 survey (survey 3) was developed using phase 2 metrics and sub-metrics receiving moderate (70–80%) or high consensus (>80%) in two contiguous priority score categories (score 4 or 5). Experts were provided with the metric name, level of consensus, and mean metric priority score from phase 2. The following prompt was used: *“Your department’s women’s professional development group (PDG) requests funding and support for the upcoming academic year. What metrics should the PDG measure to determine the success of the program? Please categorize the metrics listed below as lowest (1) to highest (5) priority in evaluating the PDG to decide whether or not you contribute funding support. The final metrics list (top 10) will be determined by mean rank scores of the metrics below.”* Participants ranked metrics by level of importance using a five-point priority scale.

#### Phase 4: Member-checking

Survey 3 was also distributed to women EM faculty and trainees across the US. The primary member prompt stated: *“Consider the following scenario: You are leading your departmental women’s professional development group (PDG) and would like to request funding and support for the upcoming academic year. As the PDG leader, what metrics do you think are important to evaluate to determine the success of the program? Please categorize the metrics listed below as lowest (1) to highest (5) priority in evaluating the PDG to support your request for funding support.”* The study question was again framed around PDG funding support because departmental specialized interest support is often requested and provided as financial or time compensation. The survey was distributed via the AWAEM and FemInEM listservs with two email reminders over four weeks.

We ranked phases 3 and 4 results by mean metric score. Metrics with a mean priority score of 4.0 or greater were sorted by consensus ranking for each group. Sub-metrics were included under the metric category assigned at phase 2. Final metric lists were compared between groups for similarities and grouped according to theme. The final metric list was used to develop a sample departmental metrics assessment tool.

## RESULTS

### Phase 1: Metric Qualitative Assessment

Of 161 experts, 39 (24%) completed the initial survey. Table 1a includes the expert panel demographics. Of respondents, 77% self-identified as ED chairs and 10% self-identified as AWAEM past or current presidents. Remaining participants (13%) self-identified their academic role as a vice chair, vice president, hospital practice chair, or vice dean. Average participant age was 57 years (SD 12.7 years); 46% of responders were female and 85% were White. Most experts (79.5%) had practiced EM for more than 15 years. The majority (87%) of participants worked at an institution with a women’s EM PDG.

Common metrics recommended by participants included the following: promotion; leadership; scholarship (described as speakership/lectures, published work, grant funding, and education-focused scholarly activity); recognition/reputation (awards, visibility); service (committee service, advocacy efforts, mentorship/ sponsorship); wellness; workplace gender equity (gender equity among faculty, presence of gender equity strategy and plan, departmental programming targeting gender equity, compensation, recruitment, retention); and PDG-specific metrics (engagement in PDG activities over time). Within each category, specific recommendations were included with a detailed assessment incorporated from responses. (See Table 2 for metrics/sub-metrics and appendix for illustrative quotes.)

### Phase 2: All Metrics Ranking

Of the 39 invited participants, 29 (74%) from phase 1 ranked 55 metrics. Table 3 lists all metrics ranked in this phase, and those with high consensus level are highlighted. We included all metrics with high or moderate consensus level in phase 3 and phase 4 surveys.

When asked to describe modifications to the metric descriptions, one participant wrote:

*… “the metrics for success for a woman who wants to make her career in operations or education or maybe a “master clinician” is different from a woman who wants to be a researcher in XX. And they all have somewhat different goals...and different metrics. The real success – and challenge – of a PDG is to support and provide skill-building/leadership training/networking/community building around the different career pathways for all women. But wellness, climate, satisfaction, and equity should be the goal regardless of career path.”*

Another participant commented:

*“Some of these metrics are controlled at the department level and may be easier to impact with the work of a PDG, whereas others are at the institutional level and it is more difficult to make change there.”*

When asked about rationale for priority ranking, one participant commented:

*“I think these things support the need for a women’s group but I’m not sure that many of these things are, or should be, the responsibility of the women’s PDG. I’d say the chair/vice chairs should stop getting funding if these things don’t improve!”*

Additional phase 2 comments are listed in the appendix.

### Phase 3: Final Metrics Ranking

Twenty-three of 29 invited participants (79% response rate) ranked 23 metrics for the final list; 52% of participants were female and average age was 46 years old. Table 3 shows the 11 key final metrics. Nine metrics had a high consensus ranking (greater than 80%) and a score greater than 4.0 on a priority scale. Two metrics had moderate consensus but were included due to their high-moderate consensus ranking (78%) and high average score (greater than 4.0). The three metrics with greater than 90% consensus included 1) gender equity strategy and plan (96%); 2) recruitment (96%); and 3) compensation (91%).

When asked about rationale for priority ranking, one participant commented:

*“Promotion and attainment of publication and grant successes are the gold standards of academic success. EM women have a flat promotion rate (REI [Rank Equity Index]-Hobgood et al, AEM) and have been for many years despite adequate numbers of women matriculating as faculty. Recruitment of women residents is declining – we must shore up this number to ensure adequate numbers of women matriculating into the discipline and subsequently becoming faculty. Our goal should be 50.5%, which is the current percentage of women medical students. In addition, the retention of women in the faculty is critically important. When women students and residents observe their women faculty leave the discipline, they question the career choice. We can attain no long-term leadership success for women without an adequate cohort and full professor status.”*

Another participant noted:

*“I think it is important to recognize that many of these factors are not for women themselves to fix. Putting the expectation that a women’s group will increase the leadership metric when there are so many factors biased against women could be an unrealistic expectation of a group like this. However, the group could put pressure on the department to develop things like an equity group. I think it’s very important to make a distinction here, lest this data be used to derail and argue against investing in such a group because it’s not effective.”*

Additional phase 3 comments are summarized in the appendix.

### Phase 4: Member-checking

The final ranking survey from Phase 3 (23 metrics) was distributed to approximately 1000 members. Table 1b includes member demographics. A total of 39 female emergency physicians completed the survey (estimated response rate 3.9%). All participants identified as female, average participant age was 42 years old, and 74% were White.

Table 4 shows the member priority metric list containing 12 key metrics. All metrics had a high consensus ranking (greater than 80%) and a score greater than 4.0. The three metrics with greater than 90% consensus included 1) compensation (92%); 2) gender equity in promotion rates among faculty (92%); and 3) gender equity strategy and plan (92%).

When asked about rationale for priority ranking, one member commented:

*“Promotion is important but takes time. AND is not a goal of every faculty member. Markers of goals accomplished makes the program personalized to the needs of the women. VERY important that gender equity is analyzed and reported by department leadership in terms of salary, bonuses, directorship/leadership positions, protected time, access to mentors/sponsors, awards, and recognition. Authorship on manuscripts – tracking gender distribution in the department – this networking often reveals major inequities in opportunities.”*

As in phase 3, member participants commented on the need to distinguish between metrics that are departmental responsibilities and those that are a PDG’s responsibility. A common theme included the need to incorporate institutional variation in metrics as each institution may ascribe different specific values to promotional criteria depending on its strengths/weaknesses. Participants highlighted the need for a comprehensive review of “successful metrics,” recommending non-traditional metrics that are equally as valuable including advocacy, community engagement, and the social impact of one’s work. Participants emphasized importance of flexible time and timelines, evaluating protected time of women vs men, discouraging use of traditional promotion timelines, and incorporating flexible scheduling support that does not impact compensation. Additional phase 4 comments are summarized in the appendix.

### Final Metric Determination

**Figure 2:**
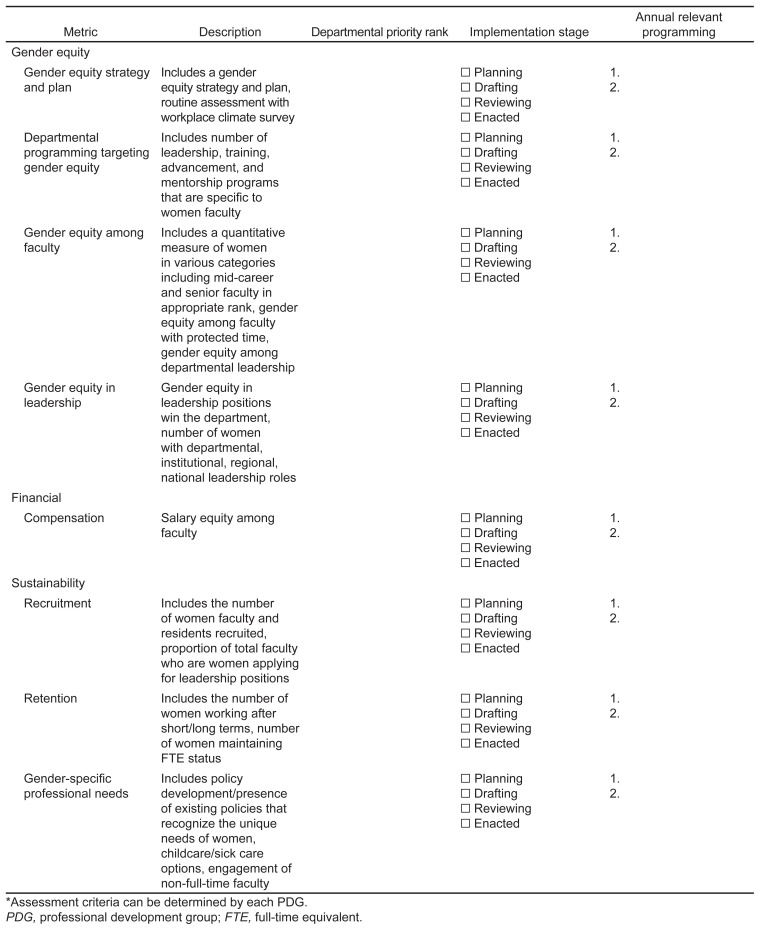

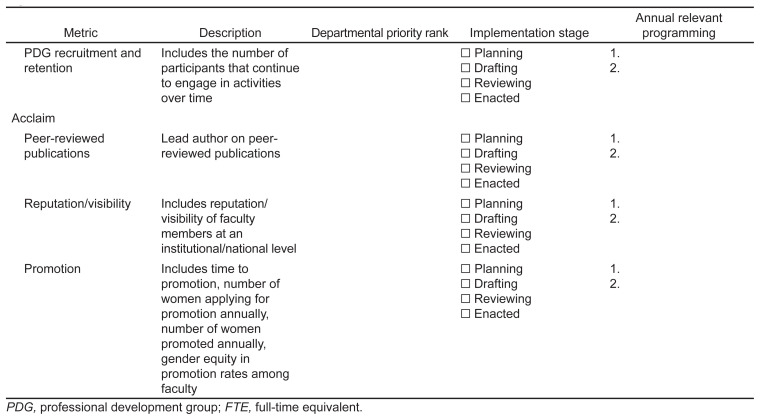
Metrics Assessment* Tool for Women’s Professional Development Groups. *Assessment criteria can be determined by each PDG. *PDG*, professional development group; *FTE*, full-time equivalent.

The following metrics achieved high consensus by experts and members: workplace gender equity; compensation; recruitment; retention; and leadership. Metrics were collated into four thematic categories: gender equity; sustainability; financial; and acclaim (Table 5) to highlight key strategic planning and intervention areas. Figure 2 displays a sample metrics assessment tool for PDGs using final categorizations and metrics.

## DISCUSSION

Our study is the first initiative to develop and rank assessment metrics for women’s PDGs in EM by expert consensus. We found that top metrics recommended by experts for departmental women’s PDGs included workplace gender equity, compensation, recruitment, retention, and leadership. Compared to experts, physician members ranked similar metric categories as most important but ranked gender equity-related metrics with higher mean scores and recruitment metrics with lower mean scores. Discussion around metric ranking centered on differentiating PDG vs departmental gender-equity responsibilities and emphasized two key themes: 1) gender equity efforts mandate departmental leadership and support; and 2) PDGs should aid leadership in addressing gender-equity gaps. Our final consensus metrics might be best targeted toward a departmental gender equity strategic plan advised by a women’s PDG.

### Departmental Gender Equity: Who Is Responsible - the PDG or Department Leadership?

A critical theme that emerged was tension between departmental versus PDG priority areas. In phase 1, many experts provided “traditional” promotion-related metrics for initial ranking, such as research grants, publications, and leadership positions. This initial metric list focused on department chair priorities and, in some ways, may lack reasonable scope for a non-funded initiative like a PDG. In later phases, the importance of delineating between evaluating a PDG versus a department on gender-related metrics became more apparent through comments and metric consensus. Participants highlighted the need for some metrics to be distinguished as departmental and institutional priorities, and the responsibility of a chair/vice chair. Respondents remarked that metrics outside the purview of a PDG could be *supported or advised* by a PDG. This is best captured by the comment that the presence of these metrics supports the need for PDG-based programming to help women improve in promotional areas, but that the department should be evaluated in these metrics, not the PDG.

The theme of the PDG as a leadership group to support and create programming to target departmental gender-equity goals is ultimately reflected in the expert metric list. Both groups highlighted a desire for targeted programming by their high rank and consensus for the “gender equity strategy and plan” metric. Gender-equity strategy and plan was the expert panel’s top ranked metric. Additional metrics, such as recruitment of female faculty and residents and departmental programming for gender equity, were also highly ranked by experts. This ranking reflects an expectation by department chairs that a PDG will focus on *efforts* to support equity but will not be measured on the *achievement* of equity.

Future work should seek to explore the expectations of departmental leadership and PDG leadership in devising a departmental gender-equity plan. While some metrics described here (ie, compensation or recruitment) might seem beyond the scope of a PDG, other metrics, such as a gender-equity strategy or gender-equity programming, would both meet a PDG’s scope and would benefit from a PDG’s expert guidance.

### Expert Panel and Member Metric Rank List Comparisons: What Matters Most?

Top ranked metric categories for both survey groups included workplace gender equity, compensation, recruitment, retention, and leadership. Metrics prioritized in our study have been described in publications on the critical role of national PDGs in academic career development.^15^,^25^ A qualitative study by Lin et al on a national EM PDG for women noted that the PDG was instrumental in helping women address the barriers (gender equity, work-life balance) and achieve metrics (awards, speakership) highlighted by our results.^15^ Similarly, a study by Pierce et al evaluating academic productivity metrics of SAEM’s Academy for Diversity and Inclusion in Emergency Medicine showed increased publications, speakership, and mentoring opportunities for leaders.^25^ National PDGs anticipate and fulfill niches for underrepresented groups in academic EM with programming and sponsorship needed for success.^26^ Priorities and goals of a PDG are consistent: building equity strategies and targeted programming are necessary to bolster women’s academic careers.

Notably, both studies reported mentorship as a successful component of a PDG. In our study, mentorship was identified in phase 1 but received moderate agreement ranking in phase 2. This finding was surprising, as previous studies indicate that mentoring programs for women are beneficial for career development.^27^ This difference in our rank position compared to previous literature may reflect the inherent nature of mentorship that exists within PDGs, and within other highly ranked metrics (eg, leadership, recruitment, retention). Alternatively, the rank position may reflect the challenge of measuring mentorship and the limited ability to measure changes in mentorship that a PDG may impact.

The high ranking of “equitable compensation” as a top PDG evaluative criterion for all participants is also interesting. “Compensation” had greater than 90% consensus and a mean score greater than 4.0. This ranking reflects the continued pervasive awareness of unequal financial compensation for women by members and leaders and the need to uphold fair pay.^2^ Compensation often falls under the purview of the department chair or institutional governance. However, all participants thought a PDG should have a role in working toward pay equity. Future discussion between the institution, department chair, and departmental gender-equity leaders should focus on PDG strategies to support equal pay initiatives. These may include transparent salary scale development, maintenance of a faculty compensation database, and/or PDG representation in institutional compensation meetings.

The member rank list also included promotion-related metrics: 1) gender equity in promotion rates among faculty; and 2) promotion. These metrics were not highly ranked by experts and were not included in the final list. Absence of promotion-related metrics from the expert rank list may reflect departmental chair belief that promotion is not the PDG’s responsibility. However, their high ranking by members may reflect member sentiment that additional mechanisms are needed to prioritize for gender equity in promotion.

### Departmental and Institutional Metric Assessment Tool for Professional Development Group

Metrics identified in our study (Table 5) can serve as an assessment tool for PDGs when developing programming and evaluating PDG initiatives (Figure 2). The tool is a guide and should be adapted to individual PDG needs and institutional/departmental goals. While the metrics described target women’s PDGs, they could be used for departmental/institutional programming. Future efforts may focus on implementation of the assessment tool to validate and refine metrics.

High rank score of equity strategy and plan suggests that a departmental gender-equity strategy and plan are essential. The details of such a plan are beyond the scope of this study, but future work could focus on key strategy and plan components, potentially incorporating highly ranked metrics identified in this study.

The potential misapplication of these metrics within a departmental faculty development plan could threaten gender equity. As noted by one participant, “Many of these factors are not for women themselves to fix.” In providing the metrics guide, our goal is to provide academic departments a framework for creating individualized gender equity targets. The PDGs cannot be strictly quantitatively measured by these metrics, as there are numerous institutional and structural barriers to attaining them. Instead, we recommend that PDG and departmental leadership meet annually to review the framework and prioritize gender-equity goals. Then, the PDG can develop programming within its effort and budgetary scope that reflects departmental goals, collect data on targeted programs, and report back to departmental leaders.

## LIMITATIONS

There are several limitations to this study. Low survey response rates for experts (~24% of the AACEM and AWAEM leaders) and member phases (~3.9% of the AWAEM and FemInEM listservs) limit generalizability. However, while small sample size may limit generalizability, the sample was large enough to reach thematic saturation.^23^,^28^ Convenience sampling may have led to overrepresentation of women in the study population, as 46% of the experts were female. Based on 2020 AAMC data, only 11% of ED chairs were women, yet 26% of our chair participants were women.^29^ Additionally, 87% of experts reported having a PDG at their institution, which may have introduced selection bias. Despite these limitations, our data reflect novel and critical themes relevant to promoting gender-equity priorities in academic medicine. Additionally, overrepresentation of women in our sample may lend accuracy to the metrics developed, as this group may be better equipped to inform achievable metrics for a departmental PDG.

The metrics and priorities described here are largely focused on faculty development and a PDG structure with significant faculty membership. However, some PDGs may focus on resident-led initiatives, and only one metric (number of women residents recruited to the residency program) was included to specifically reflect resident priorities. Future studies may examine differences in programming and evaluative metrics based on PDG leadership and membership (resident versus faculty group). Similarly, the study objective and questionnaire prompts were targeted toward supporting and funding PDGs at a departmental level, rather than institutional or national PDGs. As PDGs take a variety of forms, results may not be directly applicable to non-departmental PDGs but could serve as a guide for PDGs of other forms. Future studies may seek to better understand the time and financial resources required to attain various levels of gender-equity programming within a department.

## CONCLUSION

Experts and members recommend that academic EDs and women’s PDGs focus effort on prioritizing gender-equity programming and strategies within their institution. Equitable compensation and recruitment/retention were also highlighted as top priorities by survey participants. These top metrics represent priority domains for institutional and departmental gender-equity initiatives that are supported by a PDG. Future work is necessary to determine the optimal strategies to support PDGs’ efforts, delineate between departmental/institutional versus PDG initiatives, and establish innovative metrics that can equitably assess career advancement of all women emergency physicians.

## Supplementary Information



## Figures and Tables

**Figure 1:**
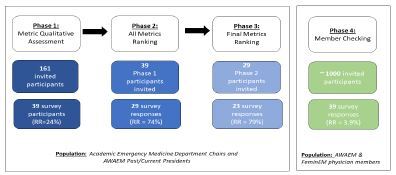
WOMENTUM study phases and participants. AWAEM, Academy for Women in Academic Emergency Medicine; RR, response rate.

**Table 1:** Study participant demographic characteristics.

1a: Expert panelist (Chairs and AWAEM Presidents demographics	

Current position characteristics	
Number of past/current department chairs, n (%)	30 (76.9%)
Number of AWAEM past/current presidents, n (%)	4 (10.3%)
Number of other, n (%)	5 (12.8%)
Gender, n (%)	
Female	18 (46%)
Race, n (%)	
White	33 (84.6%)
Hispanic/Latino	4 (10.3%)
Black/African American	1 (2.6%)
Native American/American Indian	2 (5.1%)
Asian	4 (10.3%)
Native Hawaiian/Pacific Islander	0 (0%)
Other	1 (2.6%)
Years Practicing Emergency Medicine, n (%)	
6–10 years	3 (7.7%)
11–15 years	5 (12.8%)
16–20 years	11 (28.2%)
More than 20 years	20 (51.3%)
Institutional features	
Number of participants at an institution with a women’s emergency medicine PDG	34 (87.1%)

1b: Member demographics	

Gender, n (%)	
Female	39 (100%)
Race, n (%)	
White	29 (74.3%)
Hispanic/Latino	0 (0%)
Black/African American	1 (2.6%)
Native American/American Indian	0 (0%)
Asian	9 (23.1%)
Native Hawaiian/Pacific Islander	0 (0%)
Prefer not to answer	1 (2.6%)
Years Practicing Emergency Medicine, n (%)	
< 1 year	2 (5.1%)
1–5 years	4 (10.2%)
6–10 years	9 (23.1%)
11–15 years	6 (15.4%)
16–20 years	9 (23.1%)
More than 20 years	8 (20.5%)

*AWAEM*, Academy for Women in Academic Emergency Medicine.

**Table 2:** Phase 1 responses – primary metrics and sub-metrics for ranking survey.

Primary metrics	Ranked sub-metrics
Promotion	Time to promotion
	Number of women applying for promotion annually
	Number of women promoted annually
	Gender equity in promotion rates among faculty
Leadership*	Number of women with departmental, institutional, regional, national leadership roles*
	Proportion of total faculty who are women applying for leadership positions
	Gender equity in leadership positions within the department*
Speakership	Departmental speakership
	Institutional speakership
	National speakership*
	International speakership
Published work	Author/editor of book chapter
	Peer-reviewed publications*
	Lead author on peer-reviewed publication
	Journal impact factor
	Non-peer reviewed written work
Grant funding	Number of grants applied for
	Number of grants awarded
	Type of grant received
	Role on grant received
	Gender equity in grant funding – includes proportion of women PIs on funded grants in the department
Education focused scholarly activity: *includes development or redesign of curricula*	
Awards/recognition	Total number of awards received
	Number of departmental awards
	Number of institutional awards
	Number of national awards
	Number of international awards
Reputation/visibility*: *includes reputation/visibility of faculty members at institutional and national levels*	
Committee service	Departmental committee service
	Institutional committee service
	National committee service
	International committee service
Non-committee role/title	
Advocacy efforts: *includes engagement in activities that address and/or promote a particular cause or policy*	
Mentorship/sponsorship: *includes number of mentees, regardless of gender*	
Wellness: *includes burnout and satisfaction among women faculty using validated wellness tools*	
Gender-specific professional needs: *includes policy development/presence of existing policies that recognize the unique needs of women, childcare/sick care options, engagement of non-full-time faculty*	
Gender equity among faculty*: *includes a quantitative measure of women in various categories including mid-career and senior faculty in appropriate rank, gender equity among faculty with protected time, gender equity among departmental leadership*	
Gender equity strategy and plan*: *includes a gender equity strategy and plan, routine assessment with workplace climate survey*	
Departmental programming targeting gender equity*: *includes number of leadership, training, advancement, and mentorship programs that are specific to women faculty*	
Compensation*: *salary equity among faculty*	
Recruitment: *includes the number of women faculty and residents recruited*	Number of women faculty recruited to the department
	Number of women residents recruited to the residency program
Retention: *includes the number of women working after short/long terms, number of women maintaining FTE status*	Number of women working after 1 or 2 years and long term
	Number of women maintaining FTE status
PDG recruitment and retention: *includes the number of participants that continue to engage in activities over time*	

Starred (*) metric indicate high level of consensus on survey 1.

*PI*, principal investigator.

**Table 3:** Top metrics by consensus ranking and metric score - Chair/AWAEM presidents.

Rank	Metric description	Consensus Ranking	Mean Metric Score
1	Gender equity strategy and plan	0.96	4.48
2	Recruitment	0.96	4.43
	* Number of women faculty recruited to the department*	*0.96*	*4.09*
	* Number of women residents recruited to the residency program*	*0.83*	*4.09*
3	Compensation	0.91	4.7
4	Departmental programming targeting gender equity	0.87	4.13
5	Gender equity among faculty	0.83	4.17
6	Number of women with leadership roles	0.83	4.13
7	Peer-reviewed publications	0.83	4.04
	*Lead author on peer-reviewed publications*	*0.78*	*4.35*
8	Reputation/visibility	0.83	4
9	Retention	0.83	4
10	Gender equity in leadership positions within the department	0.78	4.17
11	Gender-specific professional needs	0.78	4.09

*AWAEM*, Academy for Women in Academic Emergency Medicine.

**Table 4:** Top metrics by consensus rating and metric score - members.

Rank	Metric description	Consensus Ranking	Mean Metric Score
1	Compensation	0.92	4.71
2	Gender equity in promotion rates among faculty	0.92	4.61
3	Gender equity strategy and plan	0.92	4.41
4	Gender equity among faculty	0.89	4.51
5	Retention	0.89	4.46
	*Female faculty retention*	*0.87*	*4.35*
6	Leadership	0.89	4.41
	*Number of women with leadership positions*	*0.84*	*4.23*
7	Promotion	0.89	4.23
8	Recruitment	0.87	4.38
9	Gender-specific professional needs	0.87	4.28
10	Gender equity in leadership positions within the department	0.82	4.35
11	Reputation/visibility	0.82	4.17
12	PDG recruitment and retention	0.82	4

*PDG*, professional development group.

**Table 5:** High consensus departmental PDG metrics as evaluated by emergency medicine departmental chairs and women emergency physicians.

Gender equity	Sustainability	Financial	Acclaim
Gender equity strategy and plan	Recruitment	Compensation	Number of women with leadership roles
Gender equity among faculty	Retention		Reputation/ Visibility
Gender equity in promotion rates among faculty	Gender-specific professional needs		Peer-reviewed publications
Gender equity in leadership positions win the department			
Departmental programming targeting gender equity			

*PDG*, professional development group.
