# Supplementary material for: WOMen profEssioNal developmenT oUtcome Metrics in Academic Emergency Medicine: Results from the WOMENTUM Modified Delphi Study
Source: West J Emerg Med. 2022 Sep 12;23(5):660–71. doi: 10.5811/westjem.2022.6.56608 (PMC9541981; doi:10.5811/westjem.2022.6.56608)
Supplement: Supplementary file 1 [file wjem-23-660-s001.docx]

**Appendix: Themes and Illustrative Quotes from Survey Phases**

**Themes from Expert Panel Phase 1 Qualitative Assessment & Illustrative Quotes**

| **Theme** | **Illustrative Quote** |
| --- | --- |
| Promotion | *Promotion rates must be evaluated against an appropriate denominator - percentage or women with same number of years as male colleagues*  *Academic promotion is prestigious and often tied to compensation. Promotion is a key metric of academic and professional success.*  *Women occupy the lower ranks of faculty and are often not promoted on schedule for a variety of reasons*  *Academic rank is a widely-accepted marker of academic accomplishment and contribution*  *Rate of promotions shows efforts to mentor, support academic development of women. If there's a lag in a department, it may imply potential bias or realize/unrealized burdens/hurdles.* |
| Leadership | *Percentage of gender in key leadership groups - Vice Chairs, Executive committee, Directorships, Medical director & AMD positions, Residency leadership positions*  *Number of opportunities and programs available for leadership training and advancement. Higher weight for specific courses targeting/sensitive to gender inequities and or materials specific for gender issues. Includes personal coach, professional courses and events.*  *Number of women with leadership positions in national academic committees and societies. Evidence of national leadership is a key criterion for academic promotion at many institutions*  *We cannot solve the problem of getting women into the appropriate leadership roles unless we are intentional in every respect - offering, mentoring, retaining them in these roles* |
| Scholarship | *Invited lectures are a measure of academic productivity*  *Publications are a measure of academic productivity.*  *Grants and awards are a measure of research and academic productivity.*  *Number/proportion of women faculty with a career development award (compared to male faculty)*  *Curriculum design, clinical guideline development, educational materials*  *Developing new curricula and evaluating them is a measure of educational productivity*  *A new template for CV portfolio has been developed to show advocacy for patients, women, and under-represented minorities* |
| Recognition/ Reputation | *Awards are a measure of recognition of academic work*  *Influence throughout organization is important for personal as well as dept development*  *Subjective but reflective of personal qualities and motivation. However, can be affected by external influences both positively and negatively.* |
| Service/ Engagement | *National service is a measure of commitment to the specialty and helps one to garner a national reputation.*  *Recognition by institutional and departmental leaders for one's accomplishments* |
| Wellness | *Studies show higher levels of "burn-out" for women compared to men*  *Existence of policies recognizing unique needs of women faculty (Existence of policies for nursing (decreased RVU or academic productivity expectations, adequate time and space, etc.), childbirth (tenure clocks, etc.). Such policies are necessary to ensure women faculty can be successful*  *University sponsored or supported children's care near the work environment enables women to be present in the workplace and provides support for family (includes routine child care and sick child care). Women are the primary caregivers for many households and the brunt of sick child care falls to them regardless of profession.* |
| Workplace Gender Equity | *A women's PDG likely has the shortest term ability to ensure faculty are prepared for mid-career leadership - medical directors, program directors, etc. I would not expect the women's PDG to affect deans, chairs, etc. especially early on - this could be a long term goal*  *change university policy so part time faculty have a voice in faculty deliberations as many women choose to work part time at some time in their career and lose their voice within the academic academy*  *Variables might include: perception of inclusiveness; perception of leadership support, perception of opportunities for advancement and leadership positions; perception of mentoring, faculty development programs, perception of equity in salary; family -friendliness in policies and culture; collegiality, perception of "culture conducive to women's academic success*  *Survey of Gender based satisfaction with department, opportunities, and personal development*  *Presence of a plan to improve diversity and equity - department level, section level, and personal*  *Retention: There are many pressures on female faculty that may lead to them not remaining in the workforce or seeking opportunities elsewhere. Need annual tracking of loss/retention of women faculty. Exit interviews of departing faculty*  *Accommodations by departments for family issues: No matter how much we pretend that family life should be equally shared among partners, it's just not the case yet. Appropriate accommodations should be made given the increased family burdens that the vast majority of women continue to face.* |
| PDG Specific Metrics | PDG can be a space where women can explore other career options and bounce ideas off of each other.  Membership numbers is an indicator of willingness of people to join a highly functioning group/desire to be a part of it  satisfaction and a sense of being supported is important to demonstrate in any initiative and if successful can be a tool to mitigate burnout.  Need-based orientation: Women's groups can have all kinds of missions; it's key to see if it's actually meeting the needs of the population  Inclusiveness: Women's groups tend to be dominated by the majority group of women. Ensuring that such groups are not reproducing the problem they are purporting to tackle (lack of inclusion and advancement), it must examine and uphold equity of all kinds  Integration within the larger organization: A professional development group for women that is insular and does not provoke change in the larger institution is perpetuating the idea that women need to fix themselves in order to succeed, when in fact there are largely structural forces at play. A successful PDG should have buy in from institutional leadership. |

**Themes from Expert Panel Ranking Surveys & Illustrative Quotes**

| **Theme** | **Description** | **Illustrative Quote** |
| --- | --- | --- |
| Departmental Responsibility | Metrics that are more appropriately addressed at a departmental/institutional level, rather than being the responsibility of the PDG; the PDG can support some of these metrics but are ultimately the responsibility of the department/institution | *I think these things support the need for a women's group but I'm not sure that many of these things are, or should be, the responsibility of the women's PDG. I'd say the chair / vice chairs should stop getting funding if these things don't improve!*  *I think it is important to recognize that many of these factors are not for women themselves to fix. Putting the expectation that a women's group will increase the leadership metric when there are so many factors biased against women could be an unrealistic expectation of a group like this. However, the group could put pressure on the department to develop things like an equity group. I think it's very important to make a distinction here, lest this data be used to derail and argue against investing in such a group because it's not effective.* |
| Variability of Metrics by Institution | The value or weight of specific metrics for promotion can vary by institution; PDGs should consider institutional variability and adjust metrics accordingly | *Priority rankings for many of the metrics you listed vary widely by institution. You should know your institution and achieve the metrics accordingly.* |
| Need for novel metrics | Traditional criteria (ie for promotion) may not recognize the unique value women physicians contribute so PDGs may consider novel metrics to recognize achievements/contributions of women | *Women often bring value by their influence, involvement in mentoring, and by system innovation changes. This needs to be recognized. Traditional criteria for recognition and advancement do not fully represent the strong contributions of women faculty. There is a need to identify and use these contributions for the greater good* |
| Assessment includes raw numbers and index measurements | When assessing specific metrics, consider measuring more than raw numbers but rather using comparisons, percentages, index measurements | *Using raw numbers to describe success can be misleading. It would be better if you use an index to ensure that groups are not getting left behind. Once you achieve parity within your department it will be easier to look at raw numbers. For example, if you start with a very low number of women in the department--the numbers of faculty promoted etc will be low producing an unfavorable metric-- if you say I have 10% junior women faculty and of those 50% were promoted at the 5-year mark and that is the same as the male faculty, then you are doing well. Of course, if you only have 10% junior women faculty your recruitment metrics should be aggressive and disproportionate for hiring women!* |
| Awareness of low visibility work | Women traditionally are involved/can be encouraged to engage in low visibility work; recognizing how to balance engagement in high visibility versus low visibility work is important (ie committee work, while very valuable, can sometimes be low visibility work) | *Committee work is a time sink and can crush women faculty. It is work that is needed to keep the department and institution working but should be expected equally from male and female faculty. Committee service is valuable if it is in the area of content expertise, and it provides a valuable network for achieving one and two above, plus a network for promotion*  *There are many institutional level changes that need to be made including women's invisible professional work (ie, committee service vs being asked to be on a research project) before women will be well in medicine.* |
| Retention as a recruitment strategy | For residents and junior faculty, observing women leaving medicine may discourage them from pursuing a specialty or job. | *The retention of women in the faculty is critically important. When women students and residents observe their women faculty leave the discipline, they question the career choice. We can attain no long-term leadership success for women without an adequate cohort and full professor status.* |

**Themes from Member Checking Ranking Survey and Illustrative Quotes**

| **Theme** | **Description** | **Illustrative Quote** |
| --- | --- | --- |
| Departmental Responsibility | Metrics that are more appropriately addressed at a departmental/institutional level, rather than being the responsibility of the PDG; the PDG can support some of these metrics but are ultimately the responsibility of the department/institution | *Very important that gender equity is analyzed and reported by department leadership in terms of salary, bonuses, directorship/leadership positions, protected time, access to mentors/sponsors, awards, and recognition* |
| Variability of Metrics by Institution | The value or weight of specific metrics for promotion can vary by institution; PDGs should consider institutional variability and adjust metrics accordingly | *So much depends on the context - if you're at an institution that doesn't have a track record of external funding, applying for grants should be rewarded. If you're at a place that has multiple grants awarded, it becomes more important to look at gender equity in successful applications* |
| Need for novel metrics | Traditional criteria (ie for promotion) may not recognize valuable contributions like advocacy work, community involvement, and the social impact of one’s work | *Honestly, I think so much of this has to do with the metrics set by your department. Peer reviewed publications are the "coin of the realm" but personally I believe that there should be a more holistic view of what is deemed "success". I feel a conflict between what my department and institution cares about and what I think should be a metric.*  *Advocacy efforts can be extensive and may need additional weight* |
| Flexible Scheduling and Timelines | Metrics should incorporate flexibility timelines, de-emphasizing the need for traditional or set timelines for promotion, re-thinking what applies to full time faculty and allowing for variable FTE | *Retaining female faculty by allowing flexibility is more important than maintaining everyone at full-time*  *Timeline should be less of a metric because women bear the burden of family which always sets them back by 5-10 years. We need a system that supports them through to promotion and success even if it takes longer than their male counterparts.*  *It is important to not only develop but retain women faculty so that they can become mentors and leaders within an institution. A lot of this work requires meeting basic necessities of women who often have more "home" responsibilities than men, so that they can continue to feel that aspect of their lives does not overwhelm their work life and they continue to be part of an academic community which requires more effort for significantly less compensation.* |
| Non-clinical time | Departments should consider assessing non-clinical time provided to women faculty versus male faculty and ensure equity across the department | *How about an evaluation of protected hours compared between men and women - stratified by job descriptions/titles/roles, and race - success would be getting women more protected time to DO the work, not just more women stepping up for unpaid departmental work?*  *Non-clinical FTE may be more important to women who seek a balanced career. Thus, evaluating by full time FTE is not important. Protected time may be more important* |
| Awareness of low visibility work | Women traditionally are involved/can be encouraged to engage in low visibility work; recognizing how to balance engagement in high visibility versus low visibility work is important (ie committee work, while very valuable, can sometimes be low visibility work) | *Committee work can be problematic if the women are disproportionately being relied upon for low-visibility roles - gender equity is very important there* |
